# Comparison of machine learning and deep learning models for evaluating suitable areas for premium teas in Yunnan, China

**DOI:** 10.1371/journal.pone.0282105

**Published:** 2023-02-24

**Authors:** Guiyu Wei, Ruliang Zhou

**Affiliations:** School of Geography and Ecotourism, Southwest Forestry University, Yunnan, China; Institute for Advanced Sustainability Studies, GERMANY

## Abstract

Background: Tea is an important economic crop in Yunnan, and the market price of premium teas such as Lao Banzhang is significantly higher than ordinary teas. For planting lands to promote, the tea industry to develop and minority lands’ economies to prosper, it is vital to evaluate and analyze suitable areas for premium tea cultivation. Methods: Climate, terrain, soil, and green cropping system in the premium tea planting areas were used as evaluation variables. The suitability of six machine learning models for predicting suitable areas of premium teas were evaluated. Result: FA+ResNet demonstrated the best performance with an accuracy score of 0.94 and a macro-F1 score of 0.93. The suitable areas of premium teas were mainly located in the southern catchment of LancangJiang River, south-central part of Dehong, a few areas in the mid-west of Lincang, central scattered areas of Pu’er, most of the southern western part of Xishuangbanna and the southern edge of Honghe. Annual mean temperature, annual mean precipitation, mist belt, annual mean relative humidity, soil type and elevation were the key components in evaluating the suitable areas of premium teas in Yunnan.

## Introduction

Among the world’s most popular beverages, tea has been shown to be beneficial for health, wellness, and the treatment of chronic diseases [1]. The largest producer of tea in the world is China [2]. In addition to becoming a major cash crop in southwestern China [3], tea has also become a traditional characteristic of Yunnan’s ethnic minorities and an important economic driver, contributing to agricultural development, poverty alleviation, and increasing the incomes of tea farmers [4]. Among the 73 poor counties in Yunnan, 43 have a tea industry, making development of this industry one of the most effective ways to alleviate poverty in these areas [5]. Premium teas from ancient tree, like Lao Banzhang, Yiwu, Jingmaishan, Bingdao, Xigui, are as expensive as tens of thousands of yuan per kilogram, but their production is low and demand exceeds supply [6], thus have a high potential to expand the tea planting. However, tea farmers from poor minority countries generally have a low level of education and are less knowledgeable about the natural growing conditions of premium tea communities. They are less knowledgeable about suitable growing areas, and scientific methods for managing plantations [7]. As a result, it is necessary to develop an evaluation model that is capable of identifying the ideal growing areas for premium teas in Yunnan.

Firstly, climate variables including temperature and precipitation are important environmental variables for the cultivation of tea and other crops [8–10]. Temperatures of 17–22°C and precipitation of 1,200mm-1,500mm are the ideal conditions for the growth of Pu’er tea trees [11]. Humidity is another factor that influences a tea production area’s suitability. The suitable annual average humidity for tea planting should be more than 70% [1]. Secondly, Yunnan ethnic minorities have different methods for harvesting tea, fertilizing, irrigation, and controlling pests and diseases, so the green cropping system is also an important factor in promoting the cultivation of premium teas [12]. The artificial use of land, the price of tea, and political decisions are closely related to tea cultivation, which can result in inconsistencies in premium teas habitats [3]. Therefore, tea garden management, tea tree fertilization, and tea processing techniques are of particular importance in the cultivation of premium teas [13]. Thirdly, soil variables, including soil type, soil pH value, nitrogen, phosphorus, and potassium content within the soil are important indicators of tea leaf quality. According to Ye et al., soil PH and nitrogen, phosphorus, and potassium content were positively correlated with tea leaf quality [14]. It is recommended by Shamsheer and Ismet that soils with pH values ranging between 4.5 and 5.5 and containing 2% organic matter be planted for tea production, and soils with pH values below 4 and above 6 are incompatible with tea production [1]. Fourth, terrain variables, such as terrain conditions, can also affect the crop’s fitness [15]. According to Yang et al., the most suitable elevation for Pu’er tea trees is 1400 to 1800 meters, as well as flat and gently sloped lands with slopes of 15° or less [11]. Combined with the above variables, climate variables, green cropping systems, soil variables, and terrain variables were used as predictors of premium tea suitable areas.

Research on the prediction of suitable areas is currently focused on both flora and fauna. In terms of suitable areas of animals, various models have been used by scholars to predict the suitable areas. A study by Mudereri et al. demonstrated the feasibility of using MaxEnt (ME), random forests (RF), and support vector machines (SVM) to modify the habitat suitability of southern ground hornbills [16]. To estimate the optimal habitat for Asian horseshoe crabs in the future, Vestbo et al. used ME and SVM in order to create a niche model that could then be used to identify protected areas for Asian horseshoe crabs [17]. In their study, Lindbladh et al. used the k-NearestNeighbor (kNN) algorithm combined with information from satellite images and Swedish National Forest Survey data to predict the distribution of Aegithalos caudatus and deciduous forests [18]. A combination of five machine learning (ML) techniques was used by Muoz-Mas et al. to simulate the effects of climate change on the suitable areas of large brown trout (generalized additive models, multilayer perceptron ensembles, RF, SVM, and fuzzy rule-based systems) [19]. The findings of Su et al. have provided a better understanding of suitable habitats for Asiatic black bears and red pandas based on the use of ME and classification and regression tree (GARP) ecological niche models [20]. Zannou et al. applied and compared six statistical models (ME and generalized linear model (GLM) in a single model, generalized additive model, RF, augmented regression tree and SVM model in an integrated model) to predict the habitat suitability for ticks in order to better prevent and control ticks populations in different countries [21].

In terms of suitable areas of plants, scholars have used different models to predict the suitable areas of different pairs of plants. Angga Yudaputra et al. used an ensemble ML model with RF and artificial neural network techniques predicted endangered giant flower amorphophallus titanum in terms of habitat preference, spatial distribution and population status [22]. Mudereri et al. used RF, generalized linear model, SVM, CART, flexible discriminant analysis (FDA) and enhanced regression tree (BRT) modeling techniques and integrated models (IM) to predict current and future Striga weed distribution patterns [23]. Silva et al. analyzed the overlap of the range of Pygochelidon melanoleuca in Brazil by RF, ME and SVM algorithms [24]. Dai used the ME model to determine suitable habitat for tea tree cultivation under current climate scenarios [3]. In a prediction of the suitable areas of Tossa jute, ME and educational global climate models were used to study the effect of climate change on the distribution of Tossa [25]. Using a residual network, Yue et al. simulated the distribution of Panax Notoginseng in its suitable areas in China [26].

Furthermore, the accuracy of the prediction results for suitable areas varies considerably between models. According to Mudereri et al., the IM was the most accurate among RF, GLM, SVM, CART, FDA, and BRT models in predicting the applicability of current and future Striga weed distribution patterns [23]. In a study conducted by Hu et al., DNN models had excellent performance compared with ME, SVM, RF in predicting the habitat suitability of Framework-forming scleractinian, a deep-sea sclerenchyma coral in the Gulf of Mexico [27]. In comparison with one-dimensional convolutional networks (1D-CNN) without residual connections. Zhao et al. showed that residual connections can provide richer information about the bearing signal features and increase classification accuracy [28]. Jiang et al. introduced a convolutional attention mechanism in a deep residual network and showed that the model with the addition of the attention mechanism improved 0.65%, 0.38%, 0.62% and 0.60% in accuracy, precision, sensitivity and F1-score, respectively [29].

According to the literature review above, the study of fauna and flora habitats has gained considerable attention among scholars. However, little research has been conducted on tea’s suitable areas and the precision derived from different models varies widely. This paper will model and evaluate the suitability distribution of premium teas in Yunnan by utilizing four types of variables (climate variables, terrain variables, soil variables, and green cropping system variables) and six advanced ML methods: SVM, kNN, back propagation neural network (BPNN), Convolutional Neural Networks (CNN), Residual Network (ResNet) and FA+ResNet. It has not only been possible to identify variables associated with the quality of premium teas, but also to identify the optimal models that can be used to predict the suitable areas for premium teas in Yunnan, to provide precise guidance to tea farmers in the cultivation and production of tea, contribute to the industry theoretically and practically, and support the work of poverty alleviation in Yunnan, China.

## Study area

**Fig 1:**
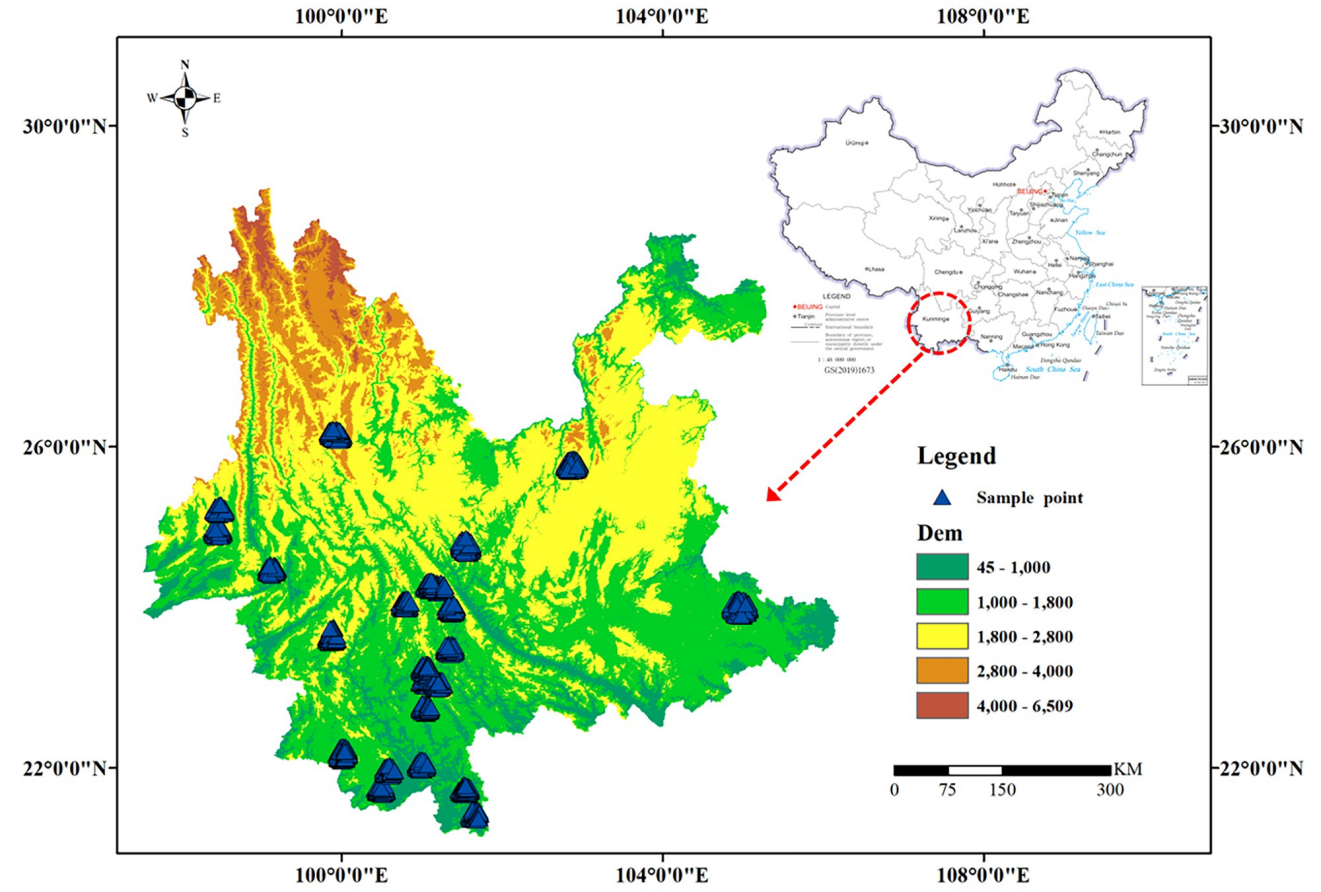
Digital Elevation Map (DEM) of Yunnan Province, China. Shape file source: republished from http://www.gscloud.cn under a CC BY license, with permission from Geospatial Data Cloud, original copyright [2022]; Own Map output: using ArcGIS 10.8 Software analysis.

Yunnan (Fig 1) is located in the southwest border of China, between 97°31′ and 106°11′ East longitude and 21°8′ and 29°15′ North latitude [30]. In Yunnan, which is a low-latitude inland region with high topography in the northwest and low topography in the southeast, the Tropic of Cancer passes through its southern end. Yunnan has 88.64% mountainous area, 87.21% mid-altitude region between 1000 and 3500 meters, and 56.46% area below 25° slope [31]. Yunnan spans six major water systems: the Yangtze, Pearl, Yuan, Lancang, Nujiang and Daying rivers [32]. Yunnan possesses a subtropical plateau monsoon climate with small annual temperature differences, large daily temperature differences, distinct dry and wet seasons, and large vertical temperature variations with altitude. The northwestern part of Yunnan has a cold climate; the eastern and central part has a temperate climate; and the southern and southwestern part has a low-heat valley climate with long summers, no winters, and rainfall in autumn [33]. In Yunnan, the average monthly temperature of the hottest month (July) varies between 19°C and 22°C, while the average monthly temperature of the coldest month (January) varies between 6 and 8°C, and the annual temperature difference is usually only 10°C to 12°C. The distribution of precipitation in Yunnan is extremely uneven geographically, with the most areas receiving 2,200mm to 2,700 mm of precipitation each year and the least receiving only 584 mm, with most areas receiving more than 1,000 mm per year [34].

## Methodology

**Fig 2:**
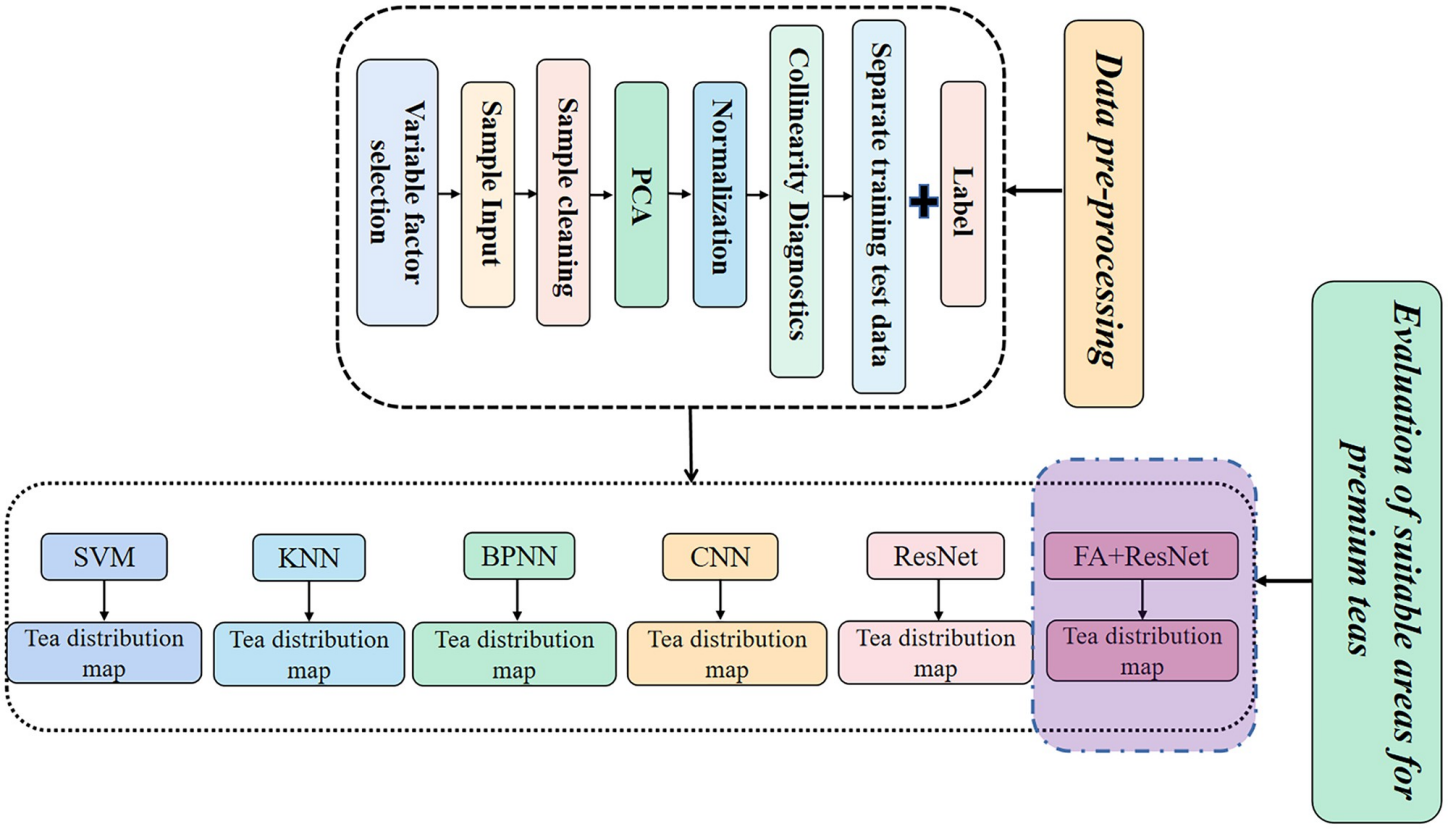
Data pre-processing and evaluation of premium tea suitable areas.

Fig 2 depicts a flowchart illustrating the entire process of evaluating the suitable areas for premium teas in Yunnan.

### Premium teas data collection

Spring tea is generally picked in March and April in Yunnan, which represents the most premium quality in the whole year. Tea leaves have higher organic matter content after winter and spring recuperation. Additionally, the quality of spring tea is superior to summer and winter teas. Normal production of spring tea accounts for 35%-40% of the year, summer tea for 30% and autumn tea for 30%. Due to this overcapacity, spring tea’s actual share of sales has increased as summer tea is often abandoned for picking, and it now occupies 65%-70% of the annual economic share of tea industry [35]. Data on premium teas for this study were collected between March 20 and April 10, 2022, and 19 tea-growing villages were visited in the field. No permits were required with the help of local farmers. We used a handheld global positioning system (GPS) with a margin of error of three meters to locate reference control points, and 23 reference data were collected with the assistance of local farmers. Reference data were collected from 14 regions including Shuangjiang County, Lincang City, Menghai County, Xishanbanna, Mengla, Xishanbanna, Lancang Lahu Autonomous County, Ning’er Hani and Yi Autonomous County, Mojiang Hani Autonomous County, Jingdong Yi Autonomous County, Zhenyuan Yi Hani Lahu Autonomous County, Tengchong City, Baoshan City, Baoshan City, Shidian County, Simao District, Pu’er City, Luquan Yi Miao Autonomous County, Dali City, Guangnan County, Chuxiong Yi Autonomous Prefecture. Using ArcMap, 120 randomly selected sampling points were created within a radius of two kilometers, with each tea tree data point as the center of a circle, resulting in 2280 samples in total. The values of each variable are then extracted to each sample point using the multi-value extraction to points in the ArcGIS spatial analysis tool to form a complete sample set of data. To minimize redundancy in distribution data during modeling, species distribution data were buffered and analyzed using ArcGIS to remove duplicate data within 1 km, ensuring that each 250 × 250 m^2^ grid contained only one tea tree distribution point [23]. Following the above screening, 2028 species distribution data were finally retained, which were distributed on different elevation gradients from 610m to 3400m above sea level.

### Variable data source and processing

Four types of variables were used in this study, mainly classified as climate variables, terrain variables, soil variables, and green cropping system variables.

Climatic variables include: annual mean temperature, annual mean precipitation, annual mean relative humidity, and cloud belt. These factors were selected because temperature and precipitation are important influencing factors for tea growth [10, 11] and the annual mean relative humidity for tea planting should not be less than 70% [1].

The annual mean temperature and annual mean precipitation data were obtained from the World Climate Information Platform (https://www.worldclim.org/), which has a resolution of 1000×1000m^2^ and a post resampling of 250×250m^2^. The annual average relative humidity data was obtained by calculating the average of meteorological station data in Yunnan Province from 2000 to 2020 and spatially interpolated, with a resolution of 250×250m^2^. Mist belt data are derived from Wang et al.’s analysis of the latitude-temperature relationship for Yunnan [36].

Soil variables include soil type, soil pH, soil nitrogen content, soil phosphorus content, and soil potassium content. Soil type data were obtained from Nanjing Soil Research Institute (https://soildata.issas.ac.cn). Soil PH, soil nitrogen content, soil phosphorus content, and soil potassium content data were obtained from the Geographic Remote Sensing Ecology Network (http://www.gisrs.cn/). The soil variables were chosen as a result of the positive correlation between the soil PH and soil N, P, and K contents with the quality of the tea [14].

The terrain variables are elevation, aspect, and slope. Terrain variables were chosen because topographic conditions also influence the range of crops that are suitable [17]. The elevation data were obtained from the digital elevation model (DEM) downloaded from the geospatial data cloud (http://www.gscloud.cn/), and aspect and slope were obtained by using the spatial analysis tool in ArcMap software with a resolution of 250×250 m^2^. Table 1 describes the predictor variables for the evaluation of suitable areas for premium teas.

**Table 1:** Predictor variables in the evaluation of suitable areas for premium teas.

Variables	Description	Units
**Climate variables**		
Annual mean temperature	/	°C
Annual mean precipitation	/	mm
Annual mean relative humidity	/	rh
Mist belt	A large area of fog that surrounds mountains or forests all year round	/
**Soil variables**		
Soil nitrogen (N)	Total amount of nitrogen in the soil	mg/kg
Soil phosphorus(P)	Total amount of phosphorus in the soil	mg/kg
Soil potassium(K)	Total amount of potassium in the soil	mg/kg
Soil pH	Acidity or alkalinity of the soil	pH value
Soil type	Soil classification is to systematically divide the soil types and their corresponding classification levels according to the differences in the quality and quantity of soil traits, so as to formulate the soil classification system	/
**Green Cropping system**		
Green cropping system	The data of the green cropping system is scored by the influence of Yunnan’s tea history and culture on tea production methods, tea picking methods, whether the tea area is a minority gathering area, whether pesticides are applied to tea trees and whether fertilizers are applied to the tea area, and other factors.	%
**Terrain variables**		
Aspect	Slope direction	Degrees
Elevation	Ground height above sea level	m
Slope	Ground steepness	%

Moreover, green cropping systems are also significant factors affecting premium tea habitat distribution [12]. The green planting system data in this paper is a comprehensive scoring of the influence of tea history and culture of Yunnan ethnic groups on tea production methods [37], tea picking methods [38], whether the tea area is a minority gathering area, whether pesticides are applied to tea trees and whether fertilizers are applied to the tea area [39] and the resolution is 250×250 m^2^.

Variable data in this study were processed using normalization, missing value processing, and principal component dimensionality reduction. The minimal data set of this study is provide in S1 Table.

The normalization process has an effect of improving the speed of convergence of the loss function, preventing the gradient explosion and improving the computational accuracy [40]. It is common practice for the input of the model to contain a variety of data of different dimensions when predicting the suitable areas for teas. In order to eliminate the influence of different dimensions on the prediction results and improve the model’s accuracy and efficiency, it is necessary to normalize the data of different dimensions. In this paper, the min-max method was used to normalize the data to 0 to 1.

Missing values can be handled in a variety of ways, including deletion, interpolation, and no processing [41]. There are 6,143,563 raster points in the prediction set data, and there are inevitably missing values when the values are extracted. Due to the fact that the raster points will ultimately be converted into raster surfaces, neither the deletion nor the no processing methods are reasonable. Therefore the interpolation method is used to complete the missing values by forward complement (’pad’) and backward complement (’bfill’) in Python [42].

Principal component analysis (PCA) is a multivariate statistical technique that reduces multiple variables to a few composite variables through the idea of dimensionality reduction. During PCA, as many characteristics of the original sample as possible are retained [26].

### Descriptive statistics on tea prices and quality

This study examined the price distribution of Yunnan tea over the study period and distinguished premium and inferior tea based on tea prices. The price of sample teas were obtained by web crawler from Taobao [43] (the largest e-commerce platform in China, and the variety and quantity of teas it sells are wide and representative) (https://www.taobao.com/) and the number and percentage of tea types in different price ranges were also calculated. In Table 2, the tea price range is between 0.8¥ and 99999¥, with a mean value of 8275.72¥ and a standard deviation of 21422.75¥. The full sample points were randomly divided into modeling and validation sets in a ratio of 8:2. In the modeling project, the statistical characteristics of the validation set and the modeling set are similar. This indicates that the validation set and the modeling set are representative of the study area for the modeling study. A validation set also isn’t involved in any modeling process, only participating in experiments as validation data. Table 3 shows the number and proportion of tea types in different tea price ranges. The tea samples are divided into 9 classes, with Class 1 representing inferior teas and Class 9 representing premium teas. The higher the price of the tea, the better the quality and vice versa.

**Table 2:** Statistics on descriptive characteristics of tea prices in the study area.

Sample grouping	Number of samples	Minimum(¥)	Maximum(¥)	Mean(¥)	Standard deviation(¥)
All sample points	2028	0.8	99999	8375.72	21422.75
Modeling set	1622	0.8	99999	8412.39	21276.23
Validation set	406	8	99999	8455.81	22014.98

**Table 3:** Number and proportion of price categories at sample points.

Price range of tea (¥)	Amount	Percentage	Label
0~100	201	9.91%	Class 1
100~200	200	9.86%	Class 2
200~500	203	10.00%	Class 3
500-~800	376	18.54%	Class 4
800~1000	221	10.90%	Class 5
1000~1500	393	19.38%	Class 6
1500~10000	170	8.38%	Class 7
10000~50000	118	5.82%	Class 8
50000~100000	146	7.20%	Class 9

### Model interpretation

#### SVM

SVM is a binary classifier whose main objective is to find the optimal classification hyperplane by using a hyperplane as a decision surface to maximize the edge between positive and negative examples [44]. In addition to reducing prediction errors, SVM also reduces the risk of overfitting. SVM can be used to solve nonlinear classification problems by mapping the data to a high-dimensional space and defining a partitioned hyperplane [45].

#### kNN

kNN is a nonparametric classification method that is widely used in a wide variety of practical classification tasks. The main theory of kNN is to find the nearest k neighbors of an unknown sample to determine its category. The implementation steps of the kNN algorithm consist of three key elements: the k-value, the distance measurement method, and the classification decision rule. Based on a grid search, the best k-value is selected using Euclidean distance as a measure of distance and the majority voting method as a classification decision method in this study [46].

#### BPNN

BPNN is one of the most widely used neural network algorithms [47], which is a supervised learning algorithm using three network layers for feedforward optimization. Its advantages include its simple architecture, ease of model construction, and fast computation [48].

#### CNN

**Fig 3:**
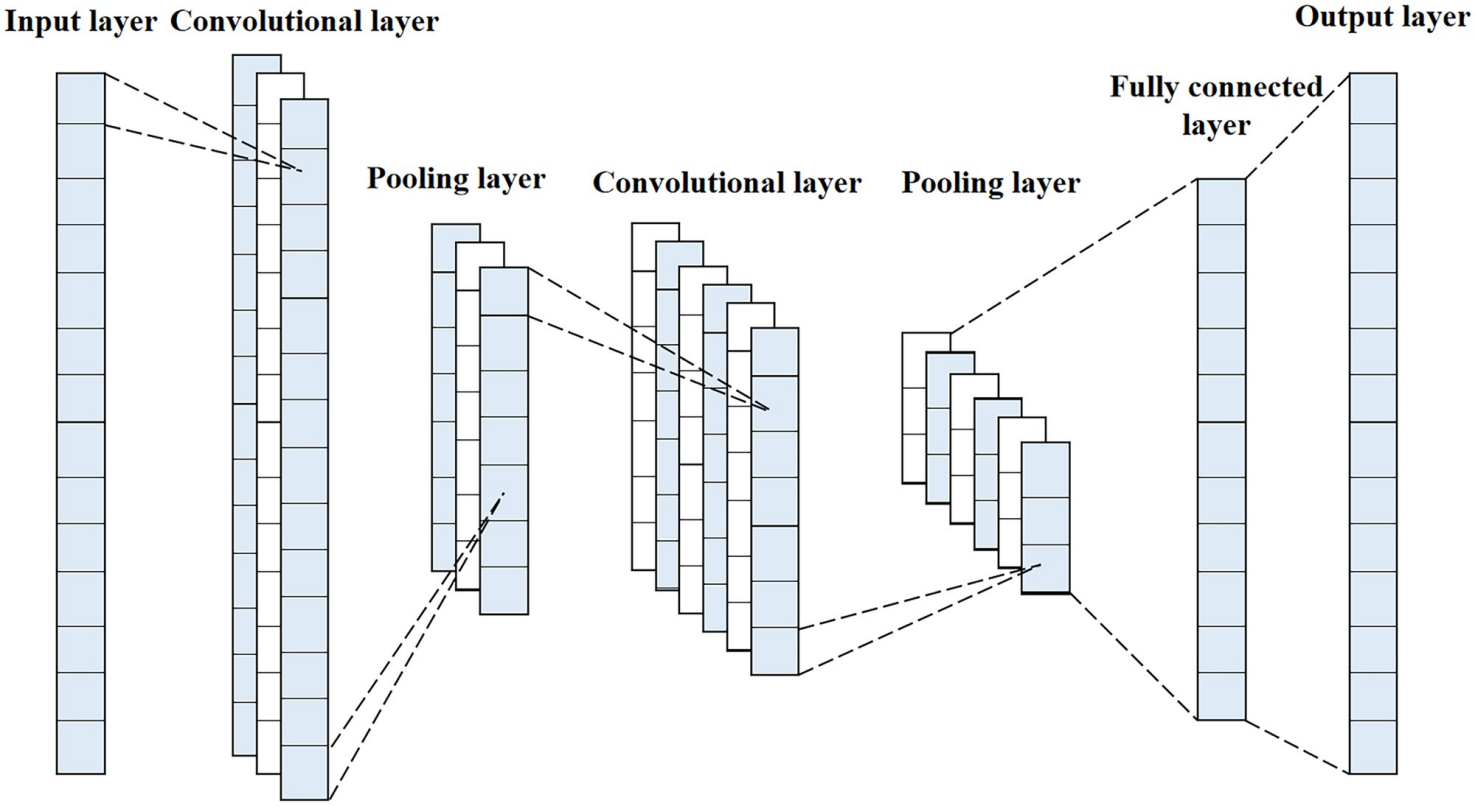
Convolutional neural network structure.

CNN is a multilayer feedforward neural network, designed for processing large-scale image or remote sensing data in the form of multiple arrays by considering local and global smooth properties. The structure of the convolutional neural network is shown in Fig 3. As compared to other deep learning structures, convolutional neural networks produce better results in areas such as image recognition and classification. CNN has local connectivity as well as weight sharing; parameter sharing can effectively minimize the problem of overfitting, and sparse connectivity allows the network to learn local features [49]. CNNs are usually composed of one or more convolutional layers, pooling layers, and fully connected layers [40].

#### ResNet block

Shallow conventional neural networks with more desirable output results may instead lead to network degradation when additional layers are added [50]. Therefore, it is difficult to fit a potential identity mapping function HX = X directly to a traditional neural network. In contrast, residual networks use residuals to convert fitted objectives into an estimation of the residuals to zero. Instead, they learn a complete output, for instance the difference between HX and X in order to achieve the identity mapping training target [51].

#### FA+ResNet

**Fig 4:**
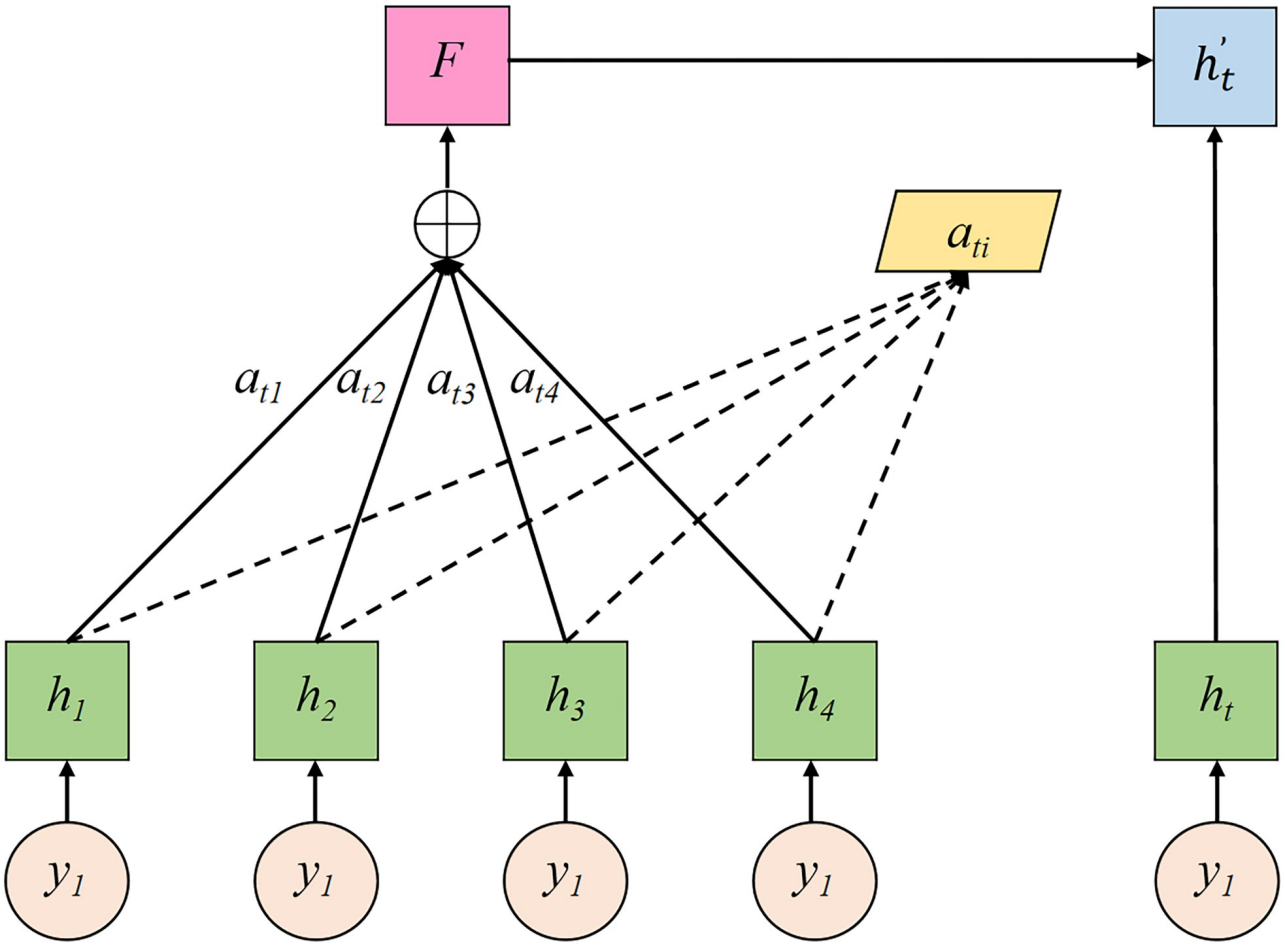
Attention mechanism unit structure.

An attention mechanism [52] is developed by simulating the attention features of the human brain, and has been initially applied to image processing [53]. The attention mechanism in deep learning assigns weights based on different features, giving greater weights to key features and smaller weights to other features, which can enhance the efficiency of processing information. Fig 4 illustrates the structure of the attention mechanism unit.

The processes of attention state transition [54] are shown in Eqs (1) to (4):

$${\text{S}}_{\text{ti}}=\text{V}\;\text{tanh}\left({\text{Wh}}_{\text{t}}+{\text{Uh}}_{\text{i}}+\text{b}\right),\text{i}=1,2,3,\cdots \text{t}-1$$ (1)

$${\text{a}}_{\text{ti}}=\frac{\text{exp}\left({\text{S}}_{\text{ti}}\right)}{\sum_{\text{k}=\text{i}}^{\text{t}}\text{exp}\left({\text{S}}_{\text{tk}}\right)},\text{i}=1,2,3,\cdots \text{t}-1$$ (2)

$$\text{F}=\sum_{\text{i}=1}^{\text{t}}{\text{a}}_{\text{ti}}\times {\text{h}}_{\text{i}},\text{i}=1,2,3,\cdots ,\text{t}-1$$ (3)

$${\text{h}}_{\text{t}}^{\prime }=\text{f}\left(\text{F},{\text{h}}_{\text{t}},{\text{y}}_{\text{t}}\right)$$ (4)

where a_ti_ is the attention weight value. y_1_, y_2_, y_3_, …, y_t_ is the input sequence. h_1_, h_2_, h_3_, …, h_t_ is the hidden layer state value corresponding to the input sequence y_1_, y_2_, y_3_, …, y_t_. h_t_ is the hidden layer state value corresponding to the input y_t_. $h_{t}^{'}$ is the final feature vector. *V*、 *W*、 *U*、 *b* are the learning parameters of the model, which continuously update with the model training process.

**Fig 5:**
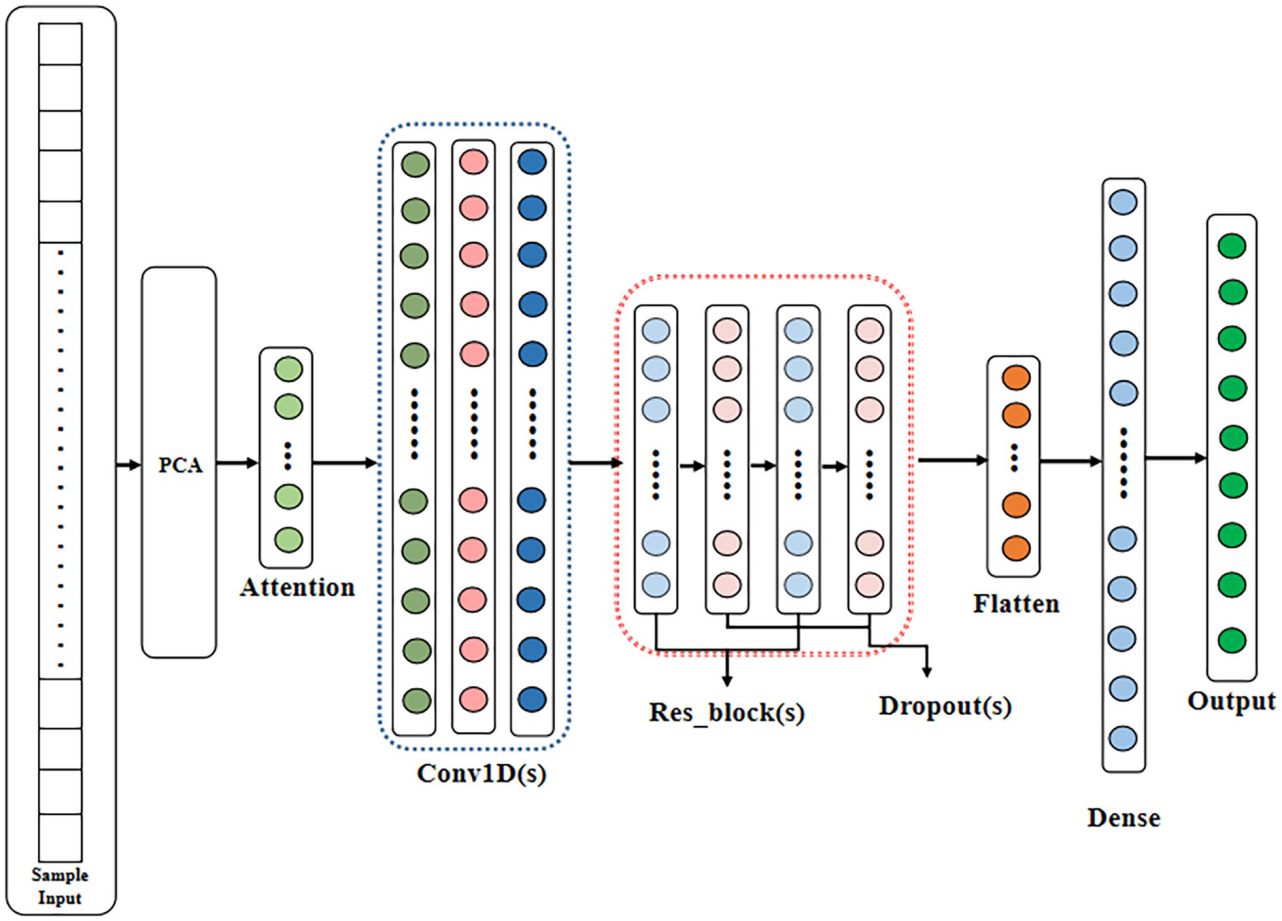
Model structure of FA+ResNet.

**Fig 6:**
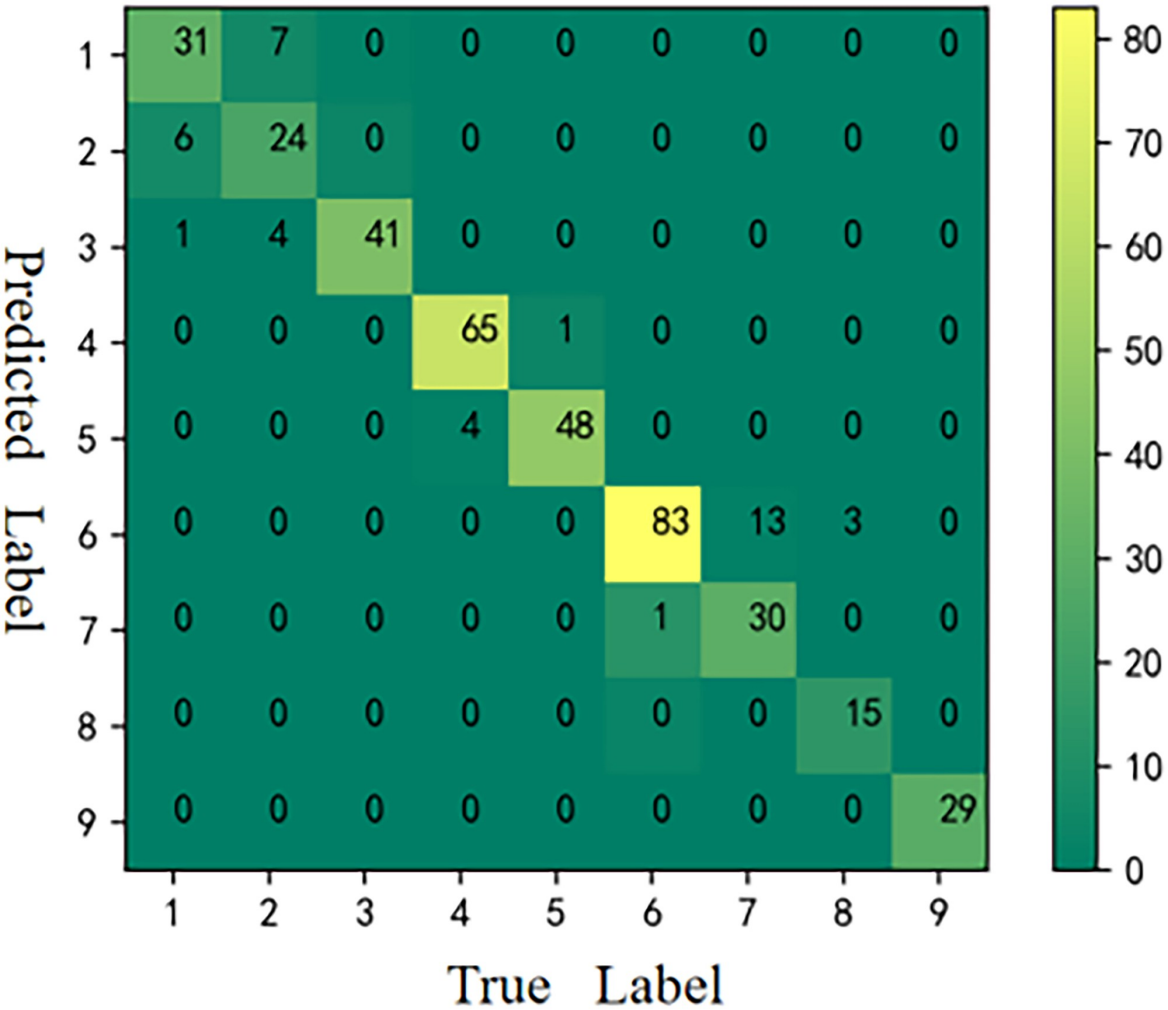
CM of SVM.

The model structure of FA+ResNet is shown in Fig 5. This is a combined model, based on residual networks, for optimizing residual network structure by adding an attention mechanism, namely feature attention (FA), to feature extraction [28]. The FA+ResNet model is based on residual network, improved residual block structure and introduced attention mechanism in feature extraction, known as the Feature Attention (FA). As can be seen in Fig 6, the FA+ResNet model mainly includes sample input layer, PCA layer, FA layer, 1D convolution layer, residual block layer, dropout layer, flat layer, fully connected layer, and output layer. The modeling processes are as followed.

- Normalize the input sample data and then perform PCA dimensionality reduction to make the data structure simpler and make the model easier to identify and process.
- Incorporate attention mechanism in feature extraction, which improves the efficiency of information processing.
- 1D convolutions are performed, and two residual blocks are then connected to form the whole residual connected part.
- To prevent overfitting, the dropout layer is added after the residual block, and the result is "flattened" by the flat layer and finally classified by the Softmax layer.

### Model assessment

#### Confusion matrix (CM)

The CM is also known as a likelihood matrix or error matrix, which is a visualization tool, especially for supervised learning. The main purpose of CM in image accuracy evaluation is to compare the classification results with the actual measured values and display the classification accuracy within itself [55, 56].

#### Accuracy and Macro-F1 score

The accuracy rate, precision rate, recall rate and F1-score are the metrics used to evaluate the classification models and directly measure the performance of the models. The greater the accuracy of the model, the better it performs, as the accuracy represents the prediction accuracy of the whole sample [56]. The calculation formula is as follows.


$$\text{Accuracy}=\frac{\text{True}\;\text{Positive}+\text{True}\;\text{Negative}}{\text{True}\;\text{Positive}+\text{True}\;\text{Negative}+\text{False}\;\text{Positive}+\text{False}\;\text{Negative}}$$ (5)

True Positive (TP): successfully predicts positive samples as positive. True Negative (TN): successfully predicts negative samples as negative. False Positive (FP): incorrectly predicts negative samples as positive. False Negative (FN): incorrectly predicts positive samples as negative.

Precision is proposed in the context of sample imbalance and is defined as the probability that the actual result is a positive sample among all the samples that are predicted to be positive, calculated as Eq (6).


$$\text{precision}=\frac{\text{TP}}{\text{TP}+\text{FP}}$$ (6)

Recall rate is the probability that a sample with a positive result will also be predicted to be positive, and is calculated as in Eq (7).


$$\text{recall}=\frac{\text{TP}}{\text{TP}+\text{FN}}$$ (7)

The F1-score hopes to find a balance point between precision rate and recall, so that the precision rate and recall results can be optimal, and the formula is (8).


$$\text{F}1-\text{score}=\frac{2\times \text{precision}\times \text{recall}}{\text{precision}+\text{recall}}$$ (8)

Macro-averaged F1 (Macro-F1 score) is the accuracy of each behavior type considered together in the case of multiple classifications. Thus it is utilized to evaluate the overall classification effectiveness of the model and is calculated as Eq (9) [57].


$$\text{Macro}-\text{F}1=\frac{1}{\text{n}}\sum_{\text{i}=1}^{\text{n}}{\text{F}}_{\text{i}}$$ (9)

## Results

### Model parameter setting

Across all six models, SVM and KNN use grid search to set parameters. The grid search method increases the accuracy and applicability of parameters in algorithms [58]. Using Python 3.8, the predictor variables affecting the distribution of suitable areas for high quality tea were input into the model and fitted to obtain the optimal distribution model. 80% of the accumulated data is used for training and 20% for testing. Below are detailed descriptions of the parameter settings for each model:

1. SVM: kernel function is "RBF", penalty factor C is 100, and error threshold gamma is 0.2.
2. kNN: the optimal parameters are: k = 3, weight = "distance", and algorithm = "auto".
3. BPNN: built in Python with a 3-layer implicit layer, an implicit layer activation function of "ReLU", an output layer activation function of "Softmax", a dropout of 0.1, a learning rate of 0.001, and an optimizer of "Adam".
4. CNN: the kernel size is 3, the step size is 1, the padding is "same", and the activation is "relu" in the 1D convolution layer. The pool size of the pool layer is 2, the padding is "same"; the activation function of the hidden layer is relu, and the optimizer is "Adam". The dropout rate is 0.1, and the learning rate is 0.001.
5. ResNet: the convolution and output layer parameters are set the same as in our CNN model. The optimizer is "Adam". The dropout rate is 0.1 and the learning rate is 0.0001.
6. FA+ResNet: "rule" and "Softmax" are the activation functions of the 1D convolutional and hidden layers, respectively. The optimizer is "Adam", the dropout rate is 0.1 and the learning rate is 0.0001.

### Results of model accuracy validation

#### Results of CM

**Fig 7:**
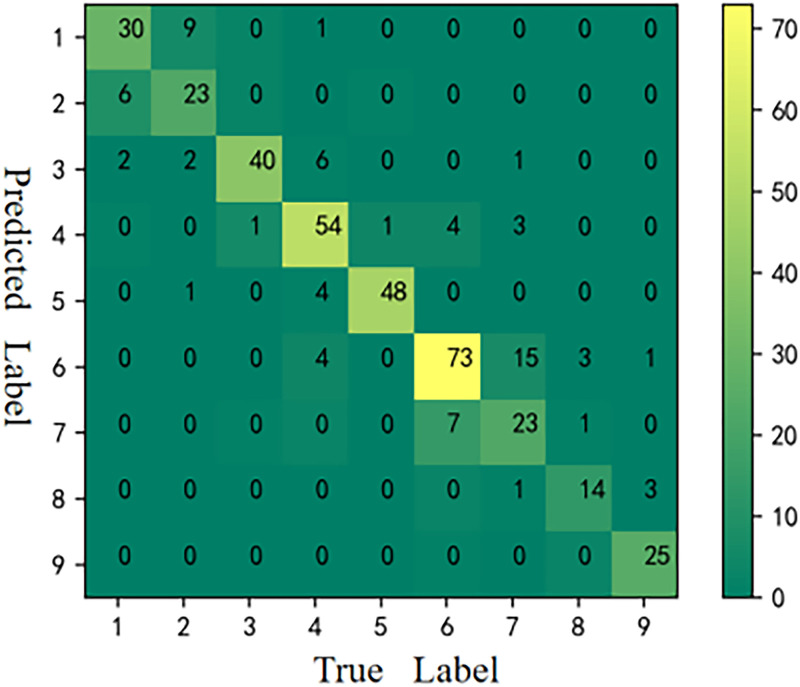
CM of kNN.

**Fig 8:**
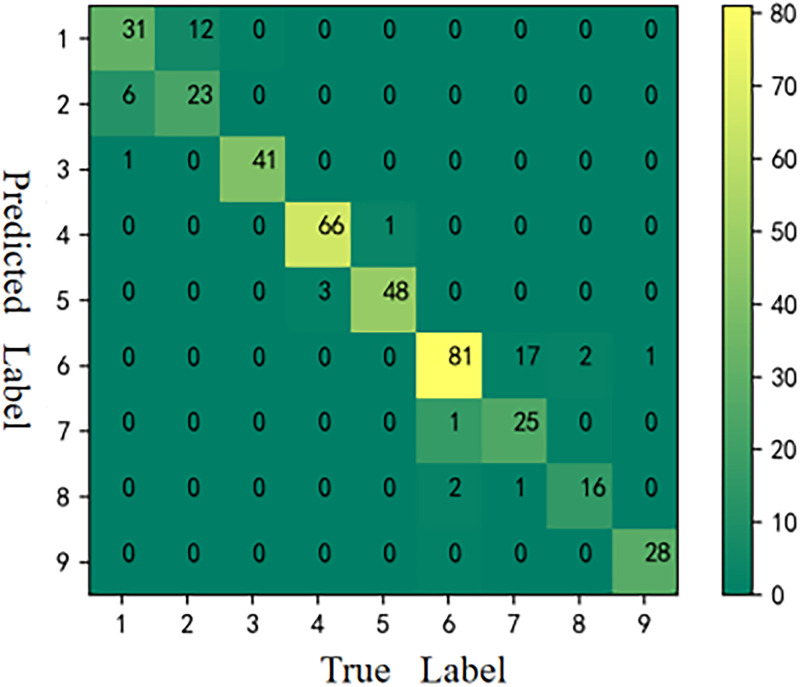
CM of BPNN.

**Fig 9:**
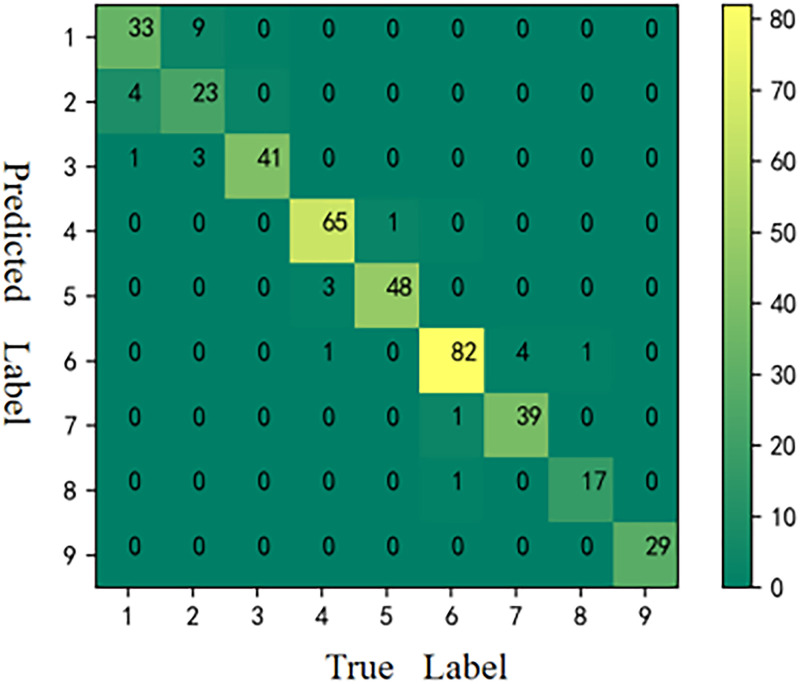
CM of CNN.

**Fig 10:**
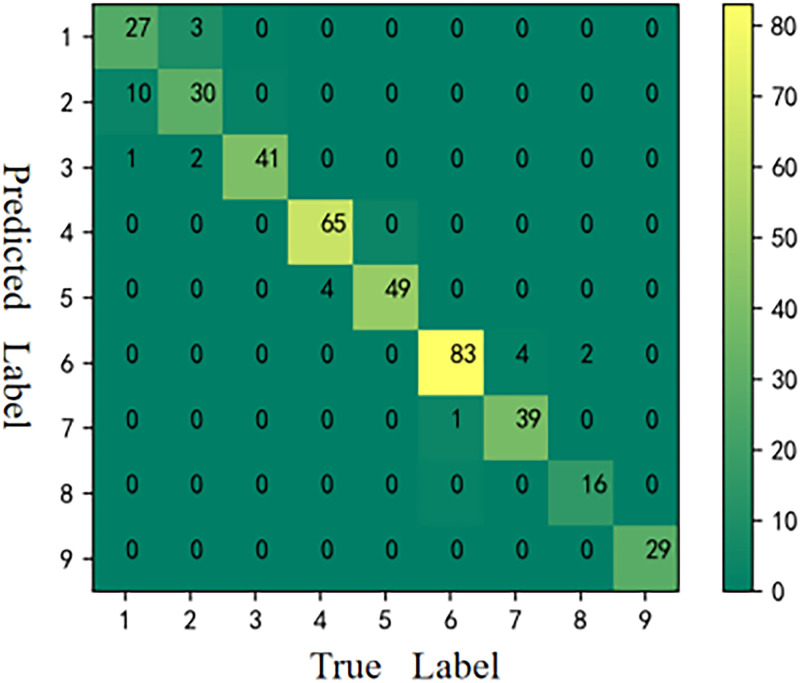
CM of ResNet.

**Fig 11:**
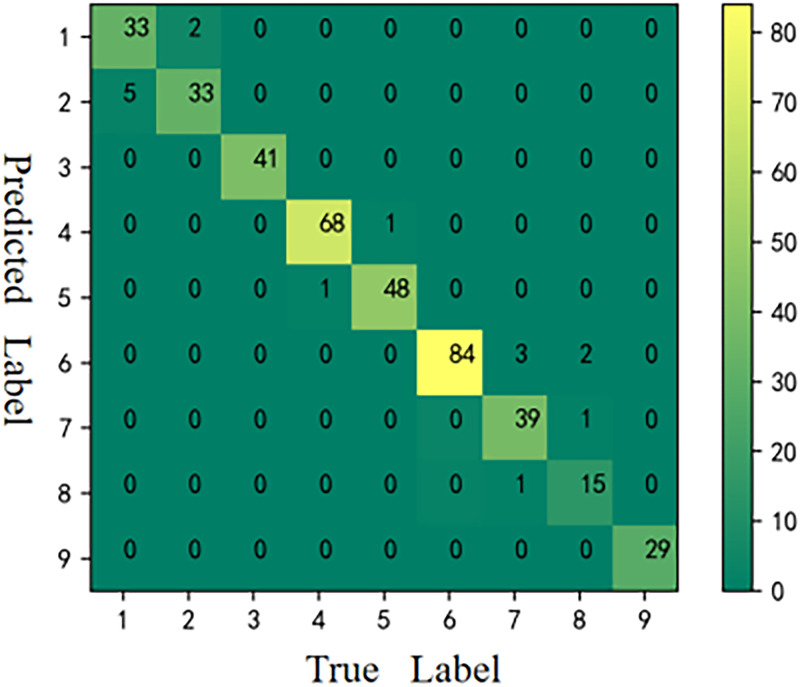
CM of FA+ResNet.

The CMs [59] of the six models is shown in Figs 6–11, respectively. The performance of the six models in predicting the suitable areas of first class tea in descending order is: ResNet<kNN<BPNN<SVM<CNN≤FA+ResNet. The performance of the six models in predicting the suitable areas of second class tea in descending order is: KNN≤BPNN≤CNN<SVM<ResNet<FA+ResNet. All six models performed well in predicting the suitable areas of second class tea, with no misclassifications. The performance of the six models in predicting the suitable areas of fourth class tea in descending order is: KNN<SVM≤BPNN≤CNN≤ResNet<FA+ResNet. The performance of the six models in predicting the suitable areas of fifth class tea in descending order is: KNN≤SVM≤BPNN≤CNN≤FA+ResNet<ResNet. The performance of the six models in predicting the suitable areas of sixth class tea in descending order is: KNN≤SVM≤BPNN≤CNN≤FA+ResNet<ResNet. The performance of the six models in predicting the suitable areas of seventh class tea in descending order is: KNN<BPNN<SVM≤CNN≤ResNet≤FA+ResNet. The performance of the six models in predicting the suitable areas of eighth class tea in descending order is: KNN<SVM≤FA+ResNet<BPNN≤ResNet<CNN. The performance of the six models in predicting the suitable areas of ninth class tea in descending order is: KNN<BPNN≤SVM≤CNN≤ResNet≤FA+ResNet. From the overall results, FA+ResNet had the highest comprehensive prediction ability.

#### Results of accuracy and Macro-F1 score

**Fig 12:**
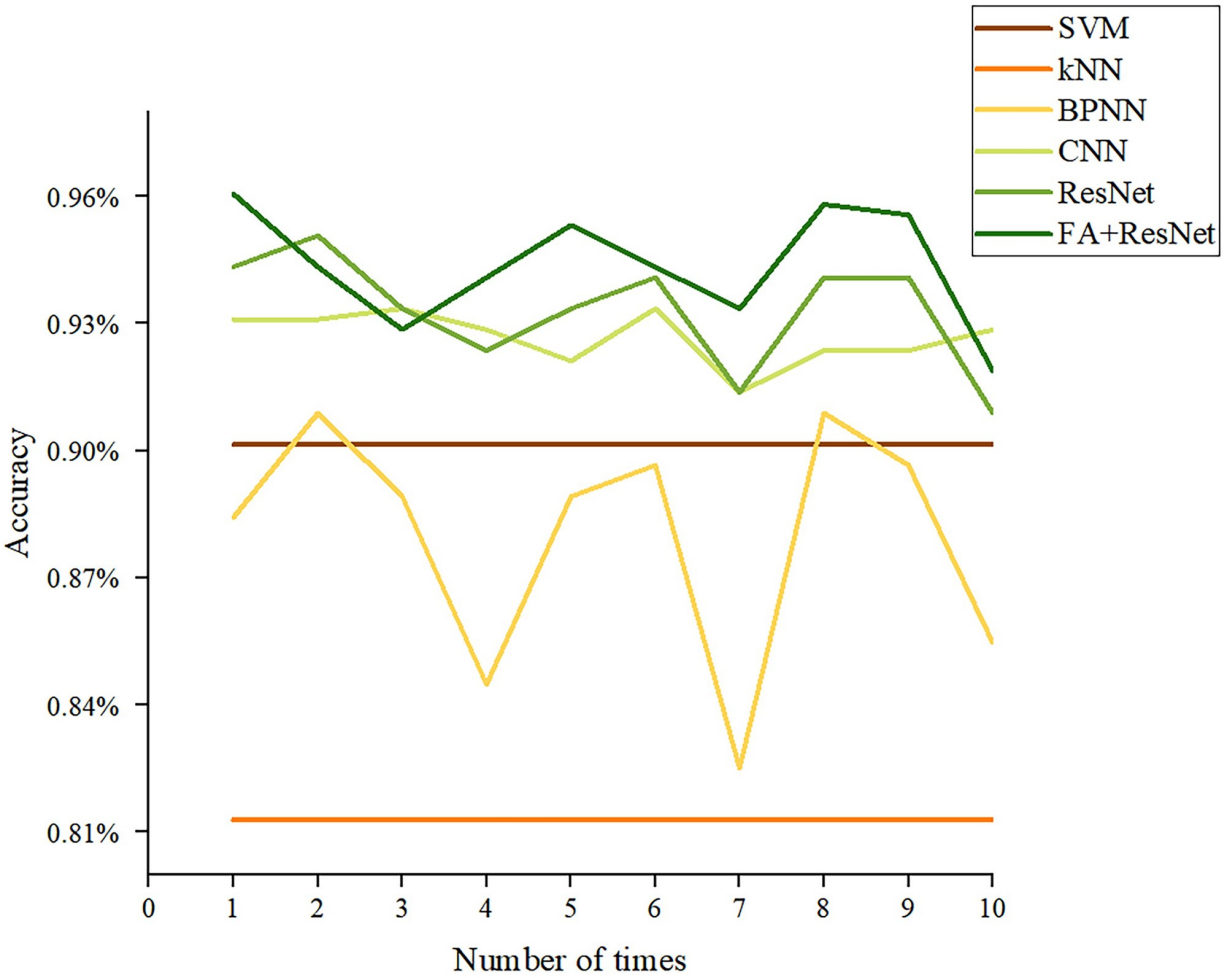
Accuracy comparisons of ten experiments with SVM, KNN, BPNN, CNN, ResNet, FA+ResNet models.

**Fig 13:**
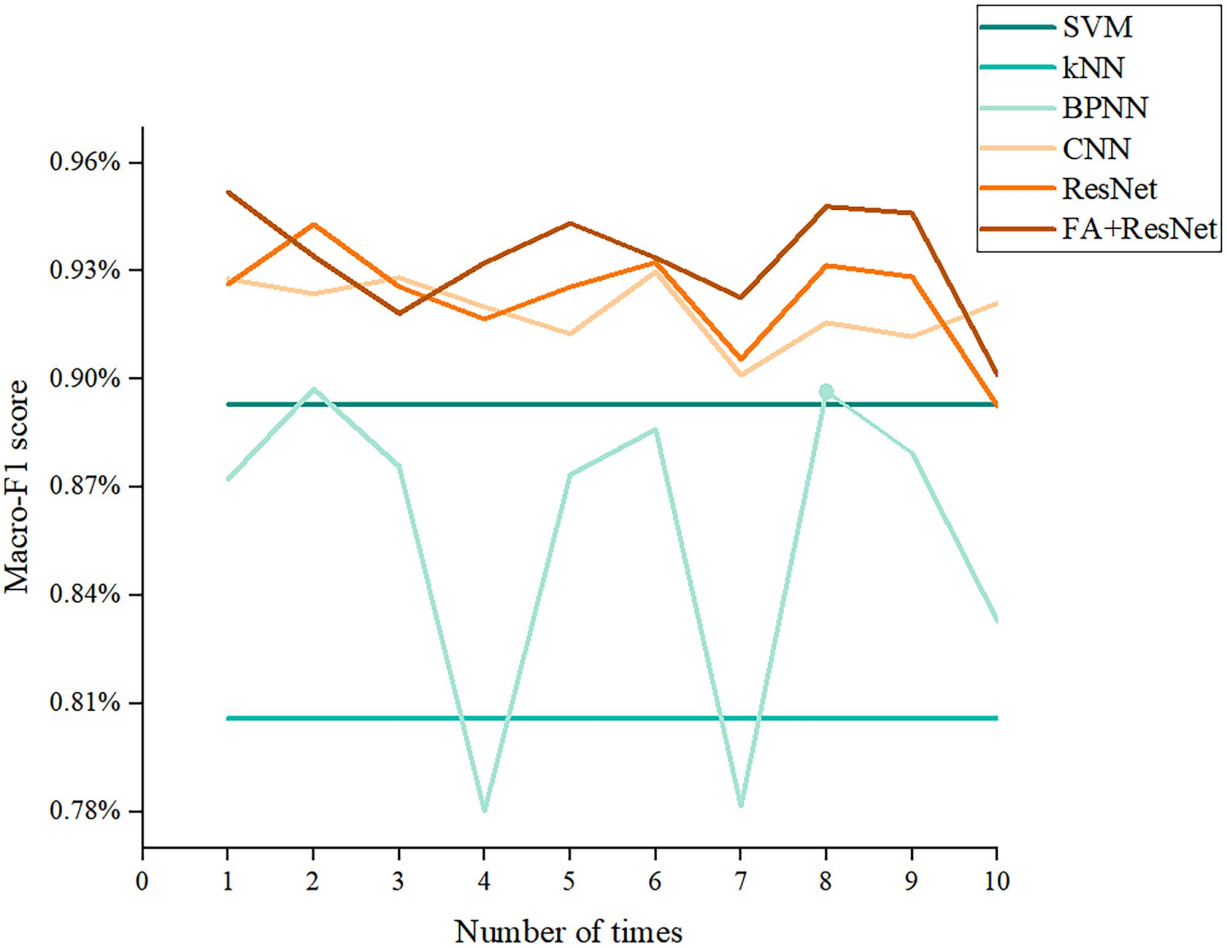
Macro-F1 comparisons of ten experiments with SVM, KNN, BPNN, CNN, ResNet, FA+ResNet models.

**Fig 14:**
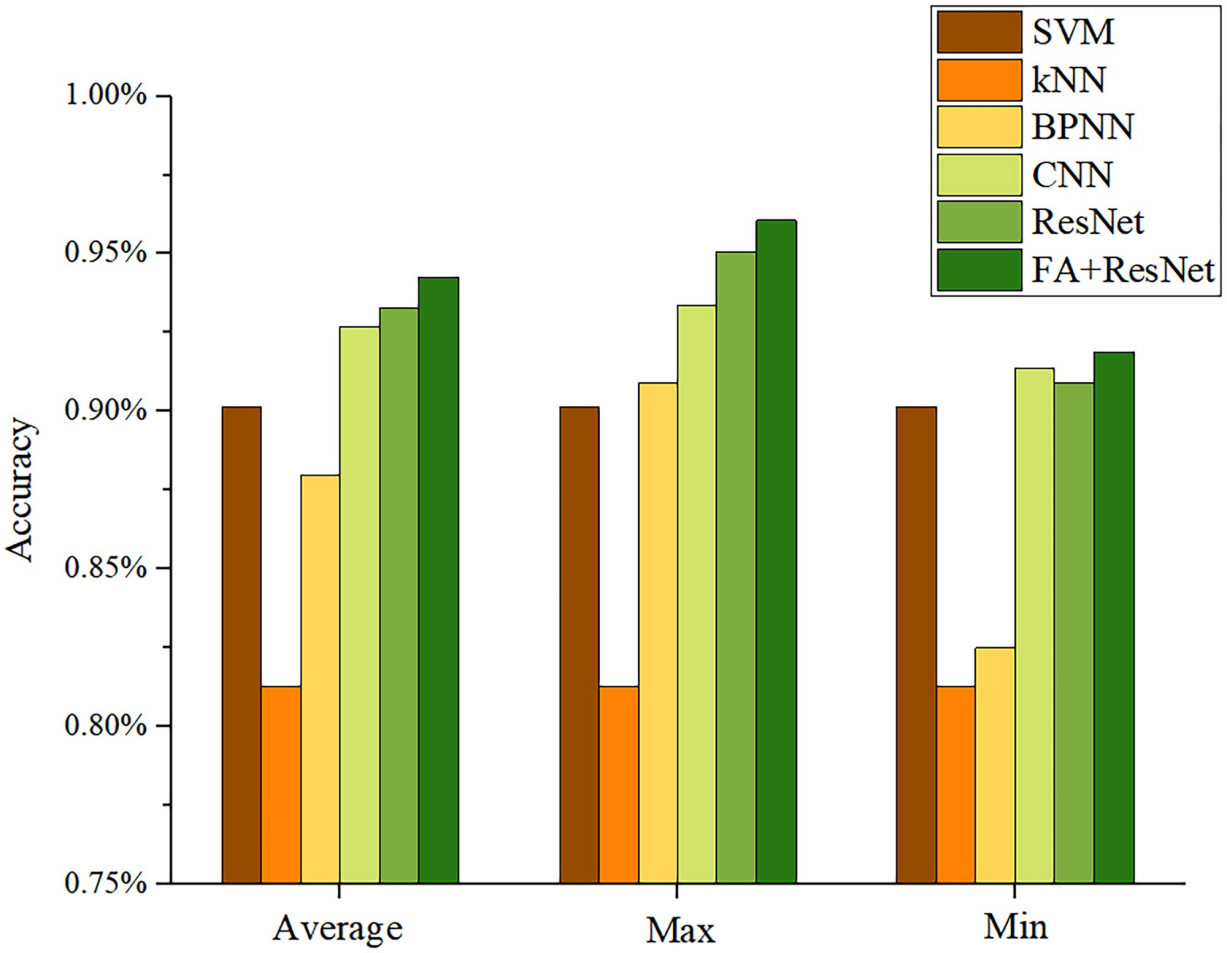
Visualization of accuracy of SVM, kNN, BPNN, CNN, ResNet, FA+ResNet models in predicting suitable areas for premium teas.

**Fig 15:**
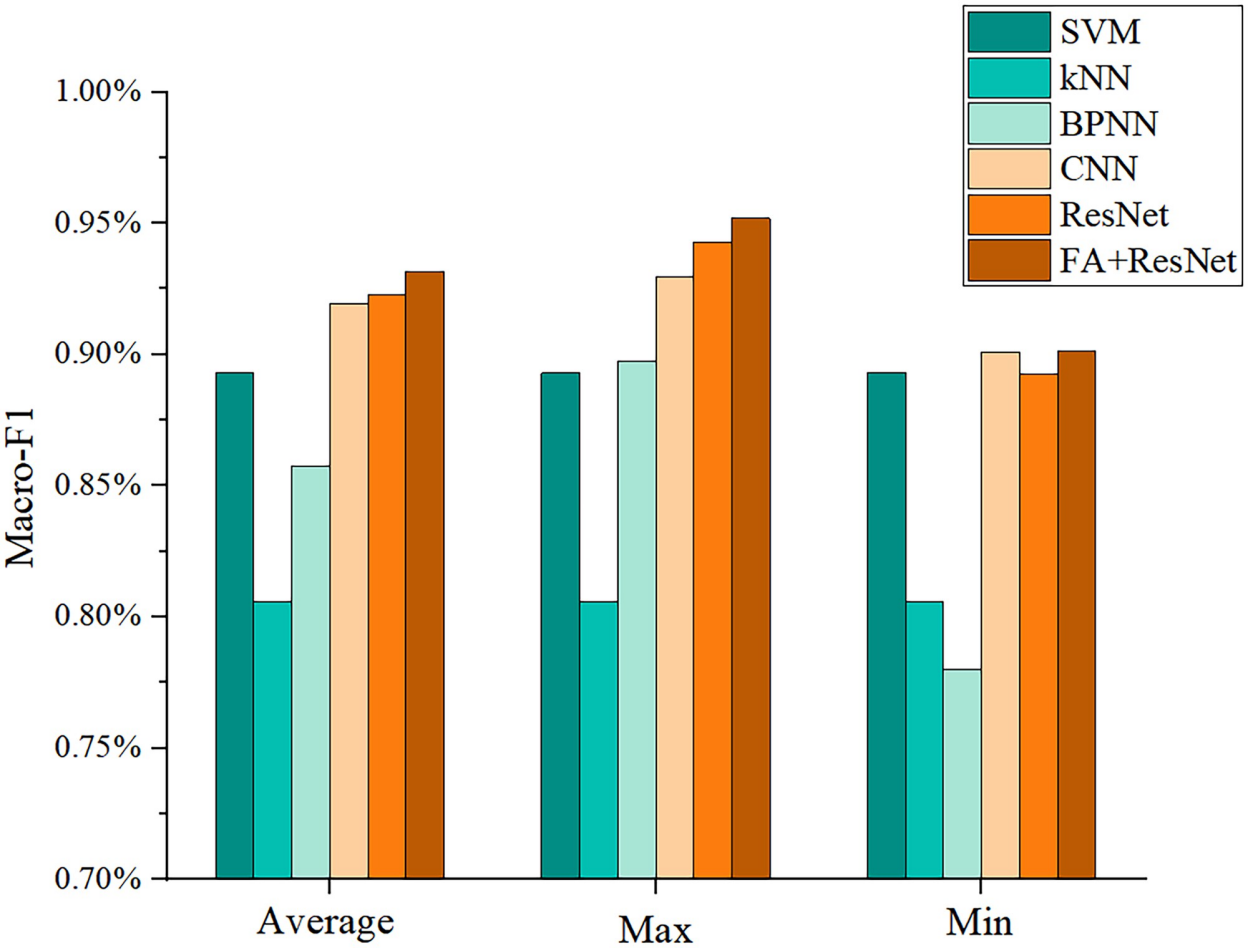
Visualization of Macro-F1 score of SVM, kNN, BPNN, CNN, ResNet, and FA+ResNet models in predicting suitable areas for premium teas.

Accuracy and Macro-F1 score are key indicators for judging the performance of multi-classification models [28]. Figs 12 and 13 describe the results of the six models obtained after ten trials for these two indicators. Both indicators show that CNN, ResNet, and FA+ResNet are more precise than BPNN, KNN, and SVM. Nevertheless, CNN, ResNet, and FA+ResNet perform differently at different stages and can’t be directly compared. Thus, the average, maximum, and minimum values of the two indicators were calculated as shown in Table 4 and Figs 14 and 15. The FA+ResNet model has both the highest accuracy and macro-F1 scores, and the overall performance of the ResNet model was slightly inferior to that of FA+ResNet. Additionally, CNN has the second best average, maximum, and minimum accuracy and macro-F1 scores after ResNet. The BPNN performs less well than the SVM, but its maximum accuracy and macro-F1scores are slightly higher than the SVM’s. The kNN model performs the worst, with the lowest average accuracy score of 81.28% and the lowest average Macro-F1 score of 80.59%.

**Table 4:** Accuracy and Macro-F1 score of SVM, kNN, BPNN, CNN, ResNet, FA+ResNet models in predicting suitable areas for premium teas.

		SVM	kNN	BPNN	CNN	ResNet	FA+ResNet
Accuracy	Average	90.15%	81.28%	87.98%	92.68%	93.30%	94.26%
	Max	90.15%	81.28%	90.89%	93.35%	95.07%	96.06%
	Min	90.15%	81.28%	82.51%	91.38%	90.89%	91.89%
Macro-F1 score	Average	89.30%	80.59%	85.76%	91.92%	92.28%	93.18%
	Max	89.30%	80.59%	89.73%	92.98%	94.29%	95.18%
	Min	89.30%	80.59%	78.02%	90.11%	89.25%	90.12%

### Prediction of suitable areas of premium teas

**Fig 16:**
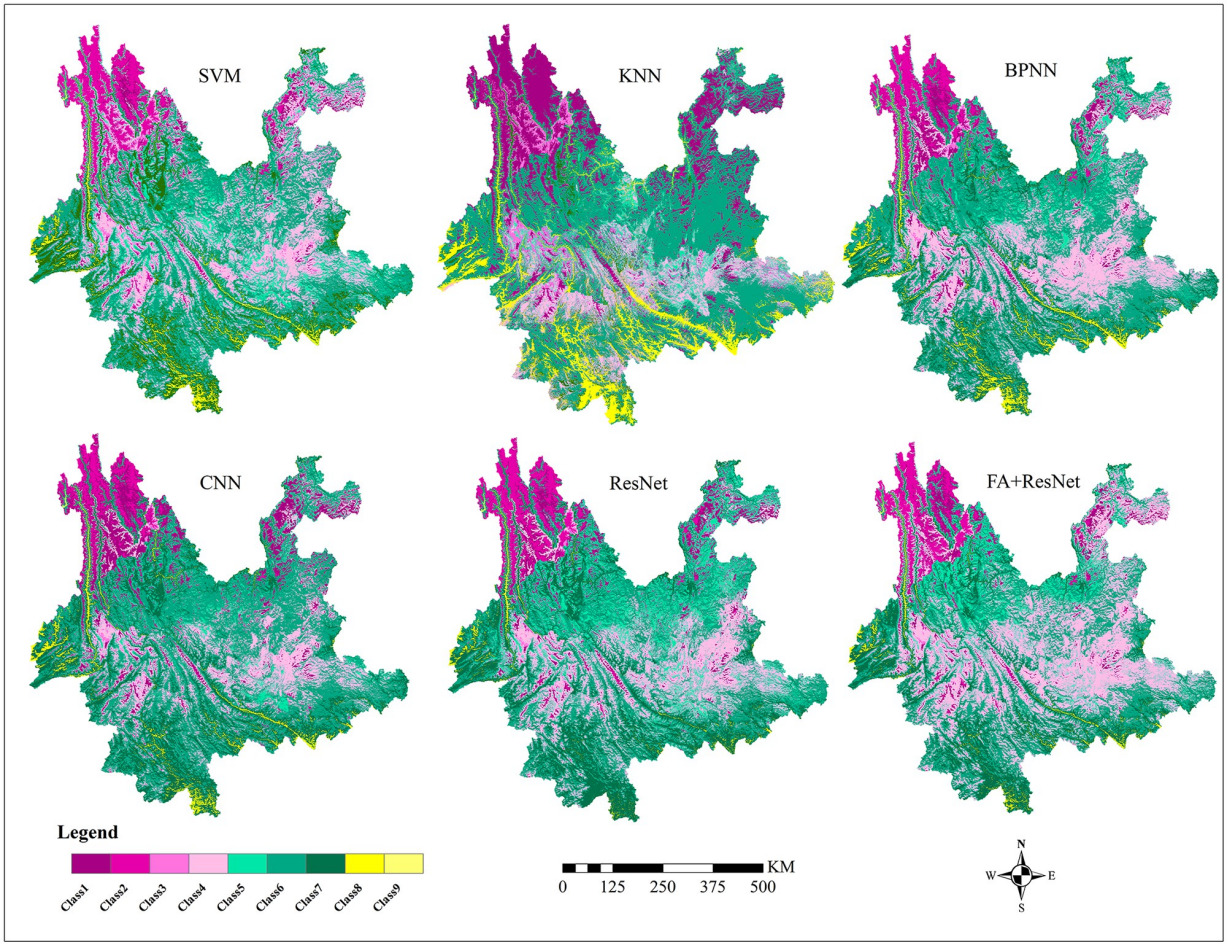
The classified predicted suitable areas of premium teas using remote sensing variables in six models: (a) SVM; (b) kNN; (c) BPNN; (d) CNN); (e) ResNet; (f) FA+ ResNet. The suitability of premium teas increase gradually from Class 1 to Class 9. Class 9 is the most suitable areas for premium teas growth, while Class 1 is the least suitable area for premium teas growth. Shape file source: republished from http://www.gscloud.cn under a CC BY license, with permission from Geospatial Data Cloud, original copyright [2022]; Own Map output: using ArcGIS 10.8 Software analysis.

Fig 16 illustrates the prediction results of six selected models, and the FA+ResNet model performed best based on the results of Macro-F1 score and Accuracy.

**Fig 17:**
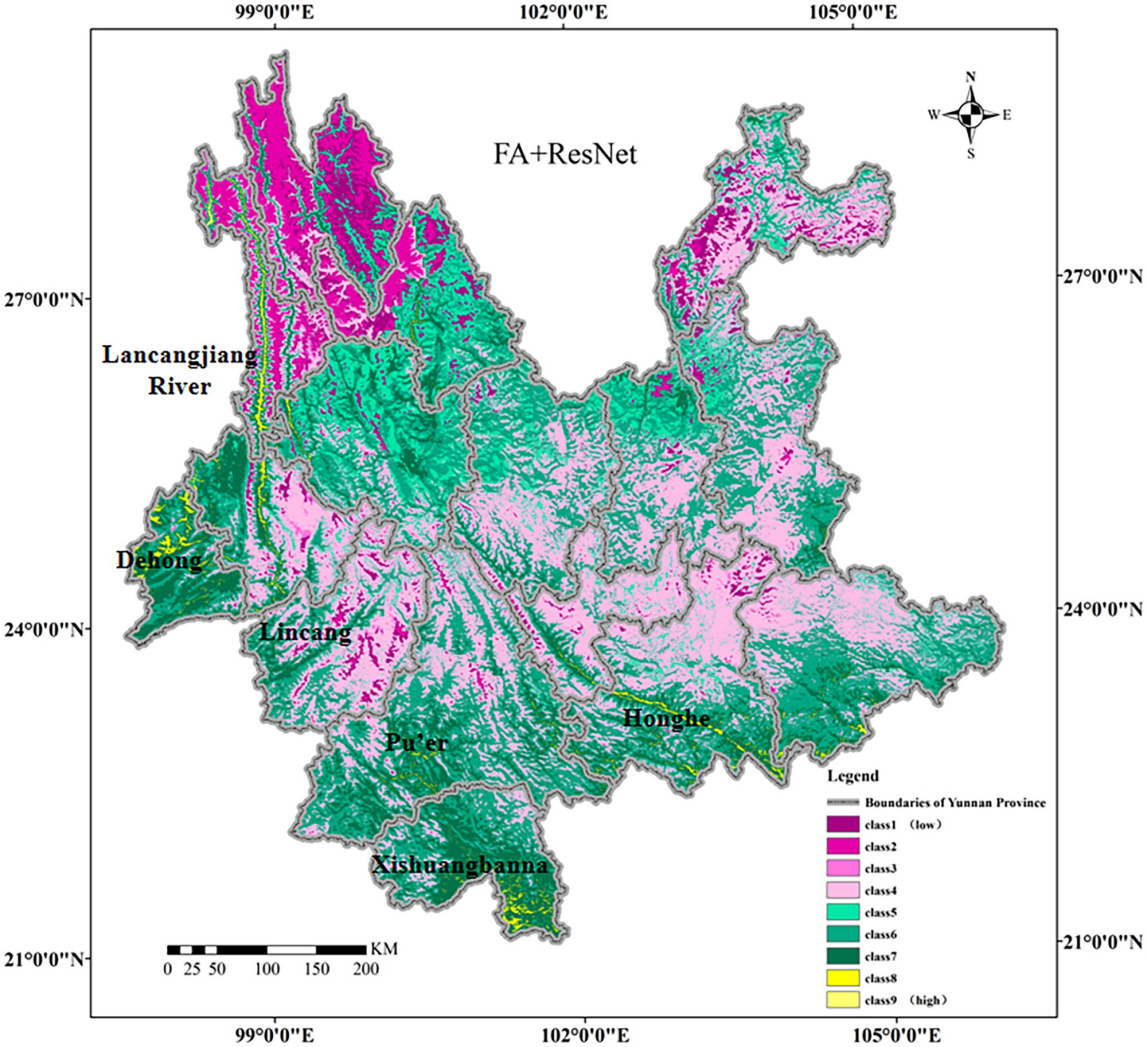
Predicted suitable areas of premium teas by FA+ResNet model. The suitability of premium teas increase gradually from Class 1 to Class 9. Class 9 is the most suitable areas for premium teas growth, while Class 1 is the least suitable area for premium teas growth. Shape file source: republished from http://www.gscloud.cn under a CC BY license, with permission from Geospatial Data Cloud, original copyright [2022]; Own Map output: using ArcGIS 10.8 Software analysis.

In Fig 17, the FA+ResNet model’s predictions for premium tea suitable areas are illustrated in greater detail and the overall probability of the distribution of suitable areas for premium teas was estimated. Firstly, the predicted suitable areas for premium teas (Class 8 and Class 9 in Fig 17) are located in the southern catchment of LancangJiang River, south-central part of Dehong, a few areas in the mid-west of Lincang, central scattered areas of Pu’er, most of the southern western part of Xishuangbanna and the southern edge of Honghe, where abundant rainfall, high humidity and fog prevail. Secondly, the northwestern and northeastern areas of Yunnan Province other than the Lancangjiang River are predicted to be low suitable areas for premium teas (Class 1, Class 2, Class 3 and Class 4 in Fig 17), due to their high altitude (mostly above 2300 m), insufficient precipitation and low temperature conditions for premium teas’ growth. Thirdly, the medium suitable areas of premium teas are mainly distributed in southwestern and central Yunnan (Class 5, Class 6 and Class 7 in Fig 17). The quality of tea in these areas decreases due to its inadequate precipitation, temperature, cultivation system, soil type, and topography.

### Variable importance

**Fig 18:**
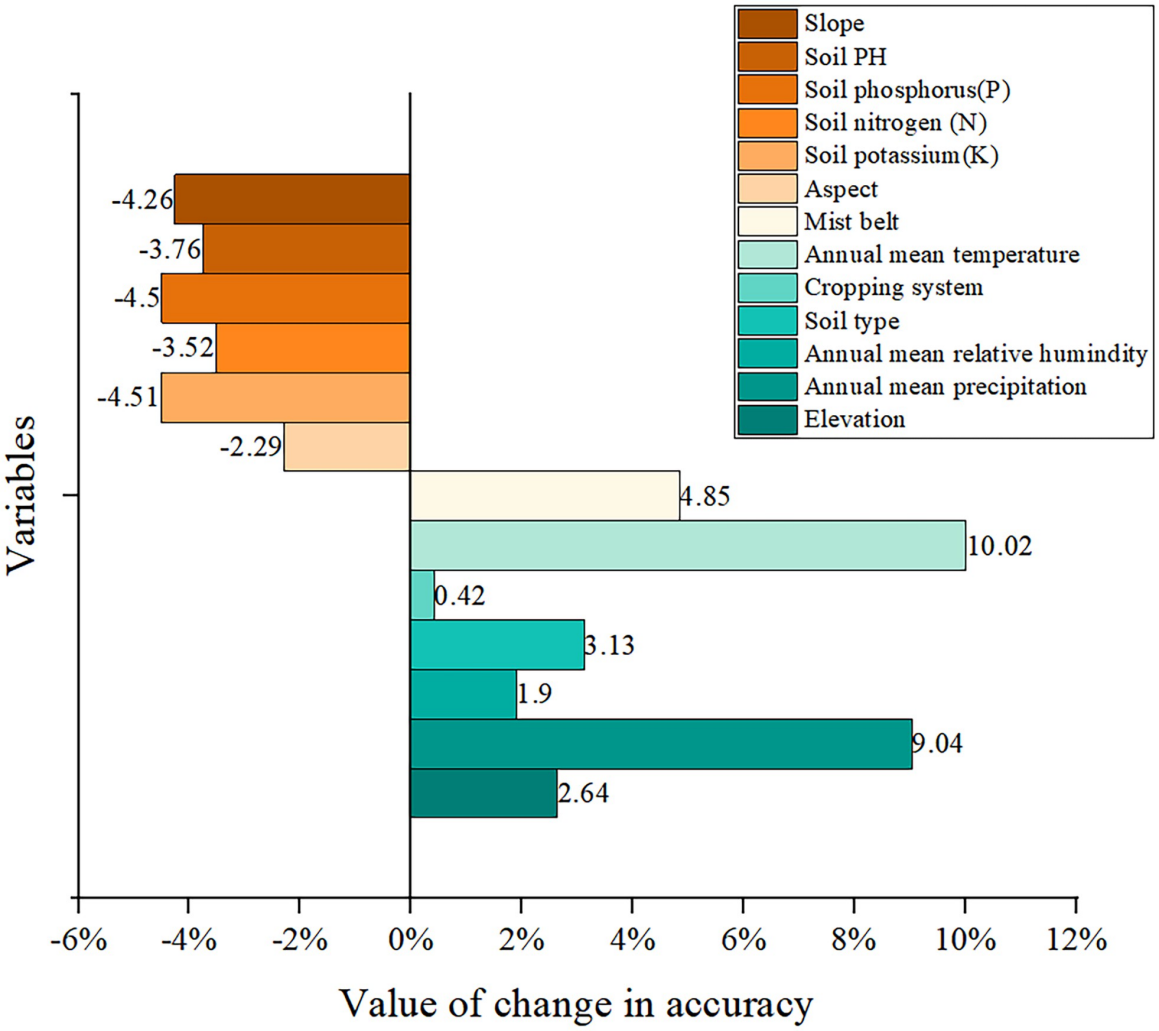
Comparison of the importance of variables in the FA+ResNet model.

By examining the importance of variables, this paper reveals the relationship between variables and suitable areas for premium tea. In our study, the FA+ResNet algorithm is used to model the remaining variables after reducing one variable at a time. Under the same conditions, the importance of one variable is calculated as the difference between its accuracy and the accuracy of a successful model. Fig 18 shows that annual mean temperature, annual mean precipitation, mist belt, soil type, and elevation are the most prominent variables. Among them, annual mean temperature and annual mean precipitation are significant factors in the corresponding predicted area. The effect of each variable on the growth of tea trees is analyzed as follows.

In terms of annual mean temperature, annual mean precipitation and annual mean relative humidity, temperature and precipitation are significant environmental variables for tea cultivation [8–10]. Temperatures of 17–22°C and precipitation of 1,100mm-1,500mm are ideal for the growth of Pu’er tea trees in Yunnan [11]. The annual mean humidity should be at least 70% for premium tea growing [1].

In terms of mist belt, one ancient Chinese saying "high mountain clouds produce premium tea" reflects the influence of mist on tea [37, 60].

In terms of soil type, different soil types have different soil pH and nutrients. In addition, organic matter, nitrogen, phosphorus, potassium and microelements are the main nutrients required for the growth of tea trees [1, 61].

In terms of elevation, the most suitable altitude for Pu’er tea trees is 1400 to 1800 m, as well as flat and gently sloping lands with slopes of 15° or less, according to Yang et al. [11].

In terms of green cropping system, most of the quality tea projection areas in this paper overlap with areas inhabited by ethnic minorities, in accordance with a study by Cao et al., which suggested that multiethnic groups such as the Brown, Yi, Dai, Hani, De’ang, and Kino have inherited intangible cultural heritage, as well as traditional production techniques and minority tea customs, through their long history of tea cultivation and production [62]. Further, the unique cultivation methods of multiethnic groups are one of the major factors contributing to the high price of premium tea. Multiethnic groups do not apply artificial fertilizer during cultivation, but rather employ a manual hand picking method that does not degrade the quality of the premium tea leaves. In addition, the ecological environment in the cultivation areas is suitable and there is little human interference with the growth of tea leaves. This ensures the distinctive flavor of the premium tea to a large degree.

According to the FA+ResNet model, slope, soil PH, P, N, K, and aspect were all statistically negative variables. It is imperative that all of them be included in the evaluation, since they are indispensable for spatially predicting suitable areas for tea [1, 11, 13].

## Discussion

In this study, it was demonstrated that deep learning methods are capable of predicting suitable areas for premium teas. Within the six models, FA+ResNet performed the best in terms of accuracy during data validation. With the use of cutting-edge artificial intelligence, FA+ResNet framework can assess crop suitability rapidly, accurately, and cost-effectively [58], which were previously difficult for quantification.

Deep learning plus attention mechanism has the advantage of obtaining more information when extracting features and preventing bias in decision making. Further, this study was more concerned with the predictive performance and accuracy of the model than the interpretability, thus FA+ResNet is the preferred approach in this context. This study has some limitations.

Firstly, this study measures the quality of tea classified by tea prices due to the fact that the market price tends to reflect the quality of tea to a large extent in most cases. However, the quality and price of teas are not completely correlated, since poor quality tea can be sold for high prices and high quality tea can be sold for high prices. Future research could choose more diverse variables to quantify tea quality.

Secondly, this study used a spatial resolution raster dataset of 250m×250m, and future studies should focus on a more refined high-resolution (i.e., sub-meter level) to detect and monitor distribution areas. Particularly, drones are suggested for monitoring the distribution area and growth of premium teas on a regular basis in order to adjust management methods as necessary.

Thirdly, in spite of the fact that premium teas in Yunnan were examined as a case study, the proposed approach can be replicated and applied to predict suitable areas for other cash crops with similar agroecological conditions.

## Conclusion

In this study, the geographical distribution pattern of different suitable areas for premium teas in Yunnan was evaluated from a combination of theoretical and practical perspectives.

Firstly, this study compared the performance of six models in multi-classification prediction of suitable areas for premium teas in Yunnan: SVM, kNN, BPNN, CNN, ResNet and FA+ResNet, based on multi-source remote sensing data. Consequently, the FA+ResNet model with the attention mechanism based on the residual network demonstrated the best performance, with the highest accuracy and macro F1-score.

Secondly, the suitable areas of premium teas in Yunnan were mainly located in the southern catchment of LancangJiang River, south-central part of Dehong, a few areas in the mid-west of Lincang, central scattered areas of Pu’er, most of the southern western part of Xishuangbanna and the southern edge of Honghe, where abundant rainfall, high humidity and fog prevail by the FA+ResNet model.

Thirdly, annual mean temperature, annual mean precipitation, mist belt, annual mean relative humidity, soil type and elevation were the significant factors in evaluating suitable areas for premium tea distribution, which corresponds to the conventional knowledge that "premium tea comes from high mountains accompanied by clouds" by local tea farmers.

As a general rule, in order to help farmers from minority groups who live in areas where premium teas are distributed escape poverty and increase their income, initiatives that enhance the management of premium tea areas should be implemented. Furthermore, researchers, policymakers, extension agents, and various stakeholders can benefit from our findings by adopting effective management programs for premium tea suitable areas.

## Supporting information

S1 Table Minimal data set. (CSV)

## Abbreviations

1D-CNN: One-Dimensional Convolutional Networks
BPNN: Back Propagation Neural Network
BRT: Enhanced regression tree
CM: Confusion Matrix
CNN: Convolutional Neural Networks
DEM: Digital Elevation Model
FA: Feature Attention
FDA: Flexible Discriminant Analysis
GARP: Classification And Regression Tree
GLM: Generalized Linear Model
GPS: Global Positioning System
IM: Integrated Models
kNN: k-NearestNeighbor
ME: MaxEnt
ML: Machine Learning
PCA: Principal Component Analysis
ResNet: Residual Network
RF: Random Forests
SVM: Support Vector Machines
VIF: Variance Inflation Factor
